# Effects of a nurse-led navigation program for first-time pregnant Korean women on maternal anxiety and childbirth confidence: a quasi-experimental study

**DOI:** 10.4069/whn.2026.05.22

**Published:** 2026-06-30

**Authors:** Suhyeon Moon, Yoon Goo Noh

**Affiliations:** 1 Department of Nursing, Changwon Hanmaeum Medical Center, Changwon, Korea; 2 Department of Nursing, Changwon National University, Changwon, Korea

**Keywords:** Anxiety, Patient navigation, Pregnant people, Self efficacy

## Abstract

**Purpose:**

This study aimed to examine the effects of a navigation program for first-time pregnant women on anxiety and childbirth confidence among primigravidas at 35 weeks of gestation.

**Methods:**

This quasi-experimental study used a non-equivalent control group pretest-posttest design. The participants were 56 primigravidas (28 experimental, 28 control) at 35 weeks of gestation or greater, who were receiving antenatal care at the obstetrics outpatient clinic of a general hospital in Changwon, Korea. The control group was recruited first and was given the standard admission education for childbirth provided in the outpatient clinic. The experimental group participated in a 3-week navigation program for pregnant women, delivered by a nurse navigator through individual face-to-face sessions. The navigator role was to assess individual needs and provide continuous, one-to-one, tailored education and emotional support to guide primigravidas throughout the childbirth process. Pretest and posttest data were collected for childbirth confidence and anxiety. The data were analyzed using descriptive statistics, t-tests, chi-square tests, Fisher exact test, and the Mann-Whitney U test.

**Results:**

The experimental group showed a greater reduction in anxiety (t=–6.15, *p*<.001) and a greater increase in childbirth confidence (z=−5.70, *p*<.001) compared to the control group.

**Conclusion:**

The 3-week navigation program for first-time pregnant women was effective in reducing anxiety and enhancing childbirth confidence during pregnancy. Findings can be used by nurses to apply individualized, nurse-led navigation as part of comprehensive maternity care for primigravidas across pregnancy, childbirth, and early postpartum periods in clinical settings.

## Introduction

Pregnancy and childbirth are unique experiences for women. Women’s needs during delivery include information, psychological and social support, respect, and safety, all of which contribute to a sense of control and empowerment during childbirth. Nurses must be able to coordinate and organize nursing care according to these individual needs [1]. Although prenatal education is routinely provided in hospitals and public health centers, studies of knowledge needs during the prenatal and postpartum periods have identified specific educational needs related to stress management, emotional stability, breastfeeding, postpartum health management, newborn care, and other maternal skills [2,3]. Compared with multiparous women, primigravidas often have greater concerns about their own health, greater fear of labor, and greater worry, expectations, and anxiety regarding the fetus. Therefore, they require accurate information and a clear understanding of childbirth [4]. First-time pregnant women also experience physical discomfort during pregnancy, the labor process, and changes in parental roles. Because these changes can represent a major life transition, multifaceted support is needed [5].

Pregnancy is accompanied by physiological and emotional changes, and a lack of information and uncertainty can contribute to maternal anxiety. Anxiety is particularly high in the third trimester as delivery approaches [6]. Maternal anxiety is an important indicator of psychological health during pregnancy and childbirth [7]. To reduce this anxiety, education provided by clinical specialists should include nursing care, information, encouragement, and support [8]. Childbirth confidence refers to the belief that one can successfully perform the behaviors needed during labor [9]. Insufficient knowledge of childbirth and a reduced sense of control can increase fear and lower childbirth confidence [10]. Conventional prenatal education is often limited to group-based, one-time information provision, which restricts its ability to continuously address individual needs. Patient navigation has been proposed as one approach to addressing these limitations, and previous studies have reported that satisfaction with prenatal care and childbirth experiences is associated with increased self-efficacy [7]. However, few studies have directly examined the effects of nurse-led prenatal patient navigation on maternal anxiety and childbirth confidence.

With increasing demand for coordinated nursing care, patient navigation can support the continuity of high-quality nurse-led care and strengthen patient empowerment [11]. In the patient navigation model, a trained navigator provides connections to healthcare services, information, community resources, and emotional support. By assessing patient needs, this model aims to improve patients’ capabilities, coping skills, and satisfaction [12]. Trained professional navigators understand their roles in two domains—continuity of care and patient empowerment—and can therefore support prioritization and decision-making more efficiently [11]. In obstetrics, the core elements of navigation include reducing barriers to prenatal care, maintaining continuity of prenatal care, linking patients to community resources, and providing psychological support. By improving satisfaction with prenatal care and childbirth experiences and helping manage anxiety, obstetric navigation may also contribute to improved obstetric and benign gynecological care [7].

Research on patient navigation in women’s health has predominantly focused on breast and gynecologic cancers [13], and patient navigation in maternal health remains at an early stage. A recent 4-week nurse-led postpartum navigation program improved continuity of care, individualized support, access to reliable advice, prompt responses to needs, decision-making support, and satisfaction [14]. In addition, patient navigation for perinatal women has been reported to improve the quality of maternal healthcare [15].

Despite the potential benefits of patient navigation in maternal care, few studies have examined prenatal patient navigation for pregnant women. Patient navigation can be applied to maternity nursing by promoting continuity of prenatal care, providing prenatal health education, supporting breastfeeding, and connecting mothers and newborns with community resources and support services [7]. Therefore, it is necessary to develop and apply a navigation program tailored to the needs of first-time pregnant women approaching childbirth and to examine its effects in this population. Accordingly, this study aimed to develop and implement a prenatal patient navigation program based on gestational characteristics and educational needs among primigravidas at 35 weeks of gestation or later who were anticipating a vaginal delivery, and to evaluate its effects on maternal anxiety and childbirth confidence.

The hypotheses of this study are as follows:

• Hypothesis 1: The experimental group receiving the prenatal patient navigation program would have lower maternal anxiety than the control group not receiving the program.

• Hypothesis 2: The experimental group receiving the prenatal patient navigation program would have higher childbirth confidence than the control group not receiving the program.

## Methods

**Ethics statement:** This study was approved by the Institutional Review Board of Changwon Hanmaeum Medical Center (No. H2024-005). Informed consent was obtained from the participants. The study was conducted in a comfortable and safe setting for participants, and voluntary participation was ensured with immediate clinical support available if needed.

### Study design

**Figure 1.**
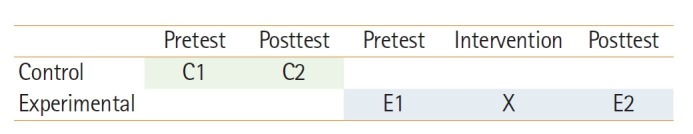
Research design. C1, E1: General characteristics, anxiety, childbirth confidence. C2, E2: anxiety, childbirth confidence.

This quasi-experimental study used a nonequivalent control group pretest-posttest design to examine the effects of a prenatal patient navigation program on maternal anxiety and childbirth confidence. Participants who received the program were assigned to the experimental group, whereas those who did not receive the program were assigned to the control group (Figure 1). This study was reported in accordance with the Transparent Reporting of Evaluations with Nonrandomized Designs (TREND) guidelines for nonequivalent control group designs [16].

### Participants

**Figure 2.**
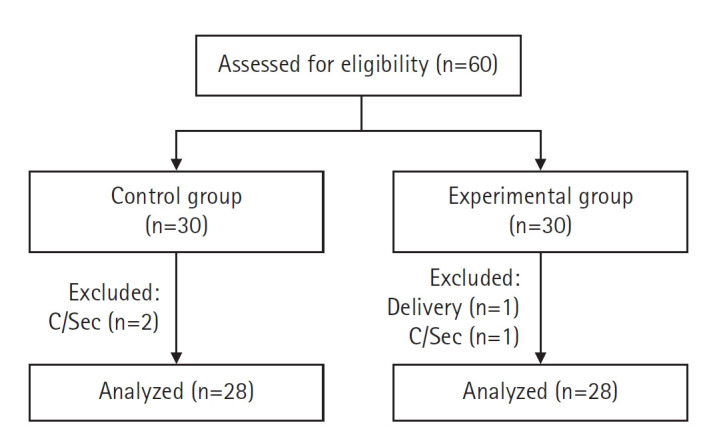
Participant flow diagram. C/Sec: Cesarean section.

The participants were pregnant women at 35 weeks of gestation or later who were anticipating a vaginal delivery and receiving prenatal care at a general hospital in Changwon. Participants were assigned to either the experimental group or the control group. The control group was recruited first through convenience sampling from May to June 2024, and the experimental group was subsequently recruited from July to October 2024. The required sample size was calculated using G*Power 3.1. Based on previous studies, with a statistical power of .80, a significance level of .05, and an effect size of .80, the minimum required sample size was 26 participants per group [8,17]. Considering potential dropout, 30 participants were initially allocated to each group, for a total of 60 participants. However, two participants in the experimental group dropped out because of delivery (n=1) or a decision to undergo cesarean section (n=1), and two participants in the control group dropped out because of decisions to undergo cesarean section (n=2). Thus, 28 participants in the experimental group and 28 in the control group were included in the final analysis (Figure 2). The specific inclusion and exclusion criteria were as follows:

#### Inclusion criteria

Participants were primigravidas at 35 weeks of gestation or later with a singleton pregnancy, a healthy fetus, and no pregnancy complications. Participants were expected by an obstetrician to have a vaginal delivery. Participants were able to communicate clearly, understand and respond to the educational content, and voluntarily provide informed consent to participate.

#### Exclusion criteria

Participants self-reported a diagnosis of an anxiety disorder or the use of anti-anxiety medication. Participants self-reported a high probability of cesarean section based on an obstetrician's clinical judgment. Participants were participating in another prenatal education program.

To prevent diffusion of the intervention effect, a time-lagged data collection approach was used. The control group was recruited and surveyed first, after which the experimental group was recruited, received the program, and completed data collection.

### Measurements

#### Anxiety

Maternal anxiety was measured using the State Anxiety Inventory (State-Trait Anxiety Inventory [STAI]-X1) from Spielberger’s STAI [18], which was modified, supplemented, and validated for the Korean population by Kim and Shin [19]. It consists of 20 items rated on a 4-point Likert scale ranging from 1 (“not at all”) to 4 (“very much so”). Negatively worded items were reverse-scored. Higher scores (possible range, 20–80) indicate higher levels of maternal anxiety. The reliability (Cronbach’s α) of the tool at the time of development was .87, and in this study, it was .91.

#### Childbirth confidence

Childbirth confidence was measured using the childbirth confidence instrument developed by Lee [20]. This 15-item instrument uses a 4-point Likert scale ranging from 1 (“not at all”) to 4 (“very much so”). Higher scores indicate greater childbirth confidence, with possible scores ranging from 15 to 60. Cronbach’s α was .89 at the time of instrument development and .83 in this study.

#### General and obstetric characteristics

General characteristics included age, education level, religion, occupation, and monthly household income. Obstetric characteristics were assessed using nine items covering gestational age, planned pregnancy, and participants’ perceptions of vaginal delivery.

### Development process of the navigation program for pregnant people

The conceptual framework of the program was constructed based on the patient navigation model [12] and the professional navigation framework [21]. The program was also designed with reference to the core elements for developing the expertise of obstetric navigators [7]. The specific components of the program were information provision tailored to individual characteristics and needs, continuity of medical services, emotional support, and linkage to community resources. The intended outcome was empowerment of pregnant women. Patient navigation programs help patients make better medical decisions and develop self-management capabilities by providing tailored information based on patient status and data. Therefore, this study designed the program to incorporate education and emotional support that addressed the specific needs of pregnant women.

In this study, one researcher, a nurse and midwife with 5 years of delivery room experience, served as the navigator and directly implemented the patient navigation program. The navigator had professional expertise in pregnancy and childbirth and comprehensively assessed participants’ physical and emotional needs, thereby supporting consistent and systematic program delivery. Before the intervention, the navigator reviewed the objectives and concepts of the patient navigation model. The program structure and weekly educational content were reviewed in advance with the goal of reducing maternal anxiety and enhancing childbirth confidence. Drawing on nursing knowledge and clinical experience related to childbirth, the navigator structured the education so that pregnancy and delivery topics could be easily understood. Supportive interventions focused on understanding participants’ discomfort and emotional needs and providing encouragement and empathy. In addition, information on public health centers and social welfare services was prepared in advance so that participants could be guided to community resources when needed.

The educational content was developed with reference to materials from the Korean Midwives Association, textbooks on women’s health nursing and midwifery [22-24], and the educational needs of expectant mothers. Educational needs were identified through interviews conducted in March 2024 with 10 mothers within 24 hours after delivery. The interviews addressed the labor process, physical changes after delivery, breathing and exercise techniques helpful for childbirth, and cooperation with medical staff. The program was structured to include information on pregnancy and childbirth and psychological support, with a focus on uncertainty about delivery and women’s self-coping strategies. The patient navigation program was then reviewed by an expert panel consisting of one obstetrician, one nursing professor specializing in women’s health, and two midwives with 30 years of delivery room experience. Based on their advice, medical terminology was revised into language that expectant mothers could understand easily, and explanations of the labor process were supplemented with concrete, real-life examples. The weekly program content was structured as follows: understanding pregnancy in week 1; experiencing the delivery room and understanding vaginal delivery in week 2; and postpartum and neonatal care and breastfeeding in week 3. The program was delivered through individualized one-to-one face-to-face sessions. Considering the period from 35 weeks of gestation to delivery, the program was designed to run for 3 weeks, with one session per week (Table 1).

In week 1, the program focused on overall understanding of pregnancy. To build rapport with the expectant mothers, the researcher introduced herself and the program, followed by a session in which each participant introduced herself and her fetus. The session then covered physical and fetal changes by gestational week, education and psychological support tailored to individual educational needs, and a question-and-answer period. The face-to-face education lasted 60 minutes. In addition, a recorded video of the educational content was sent to participants so that they could watch it at home with their spouses for 60 minutes, resulting in a total educational time of 120 minutes. Although watching the video with a spouse was not mandatory, the navigator checked whether participants had watched the video during subsequent sessions through review and questions and encouraged participants to watch it with their spouses when needed.

Week 2 consisted of experiencing the delivery room and understanding vaginal delivery. Specifically, the session included review-based feedback on the previous education, a delivery room experience, the vaginal labor process, birthing bag preparation, education and psychological support based on individual needs, and a question-and-answer period. The delivery room experience included a delivery room tour, a nonstress test, breathing and exercise techniques for labor pain management, pushing techniques, and spousal support. Practical scenarios, such as leaking amniotic fluid or absence of perceived fetal movement, were presented along with specific guidance on how participants could independently cope with and manage these situations. The face-to-face education lasted 60 minutes, and participants were encouraged to watch a recorded video at home with their spouses, resulting in a total educational time of 120 minutes.

Week 3 covered postpartum and neonatal care and breastfeeding. The session included review-based feedback, postpartum care, newborn care, breastfeeding, linkage to community resources when needed, education and psychological support according to participants’ educational needs, and a question-and-answer period. Postpartum care included uterine massage, postpartum hemorrhage, and physical changes, whereas newborn care included holding the baby, basic newborn care, and responses to neonatal emergencies. Community resources were introduced when requested by participants. Information was provided on how to use and reserve breastfeeding clinics through public health centers and community postpartum care helper services to facilitate access. The navigator reaffirmed participants’ acquired knowledge and thoughts about vaginal delivery, encouraged them to freely express their childbirth preparations and emotional states, and guided them to reflect on and articulate their personal beliefs about childbirth.

### Study procedure

This study was conducted from May to October 2024 by a midwife affiliated with the delivery room of a 700-bed general hospital in Changwon. In the experimental group, week 1 consisted of 60 minutes of one-to-one face-to-face education and 60 minutes of video-based education, for a total of 120 minutes. Week 2 consisted of 60 minutes of face-to-face education, including the delivery room experience, and 60 minutes of video-based education, for a total of 120 minutes. Week 3 consisted of 60 minutes of face-to-face education. The educational materials were created in PowerPoint (Microsoft Corp., Redmond, WA, USA), presented using a tablet and a laptop, and provided to participants as printed handouts. The control group received the standard “Admission Education for Childbirth” that had been routinely provided in the obstetrics outpatient clinic, with materials and explanations. Participants in the control group were instructed to visit the delivery room if they experienced regular contractions every 5 minutes or leakage of amniotic fluid, and they received an informational guide on items to prepare for childbirth.

Data collection for both the experimental and control groups was conducted by three trained research assistants, who were outpatient nurses, in a designated quiet area of a cafe located within the general hospital in Changwon. Both pretest and posttest surveys collected data on maternal anxiety and childbirth confidence. Data for the control group were collected from May to June 2024. Posttest data for the control group were collected in week 3 after participants received the outpatient “Admission Education for Childbirth.” To prevent diffusion of the experimental intervention, data for the experimental group were collected from July to October 2024. The pretest was administered before implementation of the patient navigation program, and the posttest was conducted after the program ended. Each survey took approximately 10 to 15 minutes. After completing posttest data collection, all participants received a small token of appreciation valued at approximately 15,000 Korean Won.

### Data analysis

The collected data were analyzed using IBM SPSS ver. 27.0 (IBM Corp., Armonk, NY, USA), with the significance level set at .05. The specific analysis methods were as follows:

(1) Participants’ general characteristics, maternal anxiety, and childbirth confidence were analyzed using frequencies, percentages, means, and standard deviations.

(2) Homogeneity of participant characteristics between the experimental and control groups was assessed using the chi-square test and Fisher exact test. Homogeneity of maternal anxiety and childbirth confidence was assessed using the independent t-test and the Mann-Whitney U test.

(3) Normality of maternal anxiety and childbirth confidence in the experimental and control groups was assessed using the Shapiro-Wilk test.

(4) To assess differences in effects between the experimental and control groups after the patient navigation program, the independent t-test was used for maternal anxiety, which met the normality assumption, and the Mann-Whitney U test was used for childbirth confidence, which did not meet the normality assumption.

## Results

### Homogeneity of general characteristics and pretest dependent variables

The mean age of the 56 primigravidas in the experimental and control groups was 31.6 years, and most participants were employed (n=38, 67.9%). Most pregnancies were planned (n=43, 76.8%), and most participants had positive perceptions of vaginal delivery (n=50, 89.3%). Pretest homogeneity testing showed no statistically significant differences between the experimental and control groups in these characteristics (Table 2). Before the intervention, the mean maternal anxiety scores were 43.93±7.39 in the experimental group and 40.68±9.47 in the control group, indicating a moderate level of anxiety. Homogeneity testing showed no statistically significant difference in maternal anxiety between the two groups.

Because childbirth confidence did not meet the normality assumption, pretest homogeneity was assessed using the Mann-Whitney U test. The mean childbirth confidence scores were 33.18±4.47 in the experimental group and 37.68±6.39 in the control group, indicating a moderate level of childbirth confidence. A statistically significant difference was observed between the two groups (z=–3.08, *p*=.002).

Because the sample size in each group was less than 30, normality tests were conducted for the dependent variables. Maternal anxiety in the control group (*p*=.246), childbirth confidence in the control group (*p*=.098), and maternal anxiety in the experimental group (*p*=.631) met the normality assumption. However, childbirth confidence in the experimental group did not meet the normality assumption (*p*=.033).

### Hypothesis testing

An independent t-test was used to analyze between-group differences in changes in maternal anxiety scores. In the experimental group, the posttest score was 35.39±6.30, representing a decrease of 8.54±7.40. In the control group, the posttest score was 44.50±7.63, representing an increase of 3.82±7.64. The between-group difference in score changes was statistically significant (t=–6.15, *p*<.001), supporting Hypothesis 1 (Table 3).

The Mann-Whitney U test was used to analyze between-group differences in changes in childbirth confidence scores. In the experimental group, the score increased by 6.5 points to a posttest score of 39. In the control group, the score decreased by 2.5 points to a posttest score of 33.5. The between-group difference in score changes was statistically significant (z=–5.70, *p*<.001), supporting Hypothesis 2.

## Discussion

This study developed and implemented a prenatal patient navigation program for primigravidas at 35 weeks of gestation or later and examined its effects on maternal anxiety and childbirth confidence before delivery. Changes in maternal anxiety and childbirth confidence differed between the experimental group, which received the intervention, and the control group, which did not. These findings indicate that the prenatal patient navigation program led by a nurse with midwifery qualifications developed in this study was beneficial for first-time pregnant women preparing for childbirth.

Maternal anxiety decreased after the intervention in the experimental group that participated in the prenatal navigation program, whereas anxiety increased in the control group. The between-group difference in change scores was statistically significant. Because few previous studies have examined prenatal patient navigation programs, direct comparison is difficult. However, the anxiety-reducing effect observed in this study can be interpreted in the context of previous studies of prenatal education programs. In particular, the findings are supported by previous studies showing that experience-oriented prenatal education reduced maternal anxiety [17] and that anxiety decreased significantly when a dedicated nurse directly delivered the program [13].

These previous studies reported that anxiety was reduced not only through information provision but also when pregnant women directly experienced the delivery environment and process and received continuous support and education from a consistent provider. The prenatal patient navigation program in this study had similar features to experience-oriented prenatal education because a dedicated navigator continuously provided interventions that included prenatal education and a delivery room experience. In addition, this navigation program differed from conventional prenatal education because it did not provide the same standardized content to all participants. Instead, the navigator continuously assessed each participant’s anxiety-related factors and responses and adjusted the educational content and emotional support accordingly. This tailored approach may have helped women in late pregnancy better understand and accept childbirth and the postpartum period, thereby contributing to reduced maternal anxiety.

Pregnant women in the perinatal period commonly experience worry and anxiety about pregnancy, delivery, the postpartum period, and related outcomes. These concerns increase during late pregnancy, and one study reported that 93% of women in the third trimester experienced moderate or higher levels of childbirth anxiety [25]. Perinatal maternal anxiety is recognized as a factor that negatively affects pregnancy, delivery, and maternal and child health [26]. Because maternal anxiety can affect both pregnant women and their children, prenatal care should include proactive identification of women with anxiety and systematic interventions to manage maternal anxiety.

A literature review categorized the causes of perinatal anxiety as psychological, social, environmental, and obstetric factors, indicating that anxiety has multiple contributing causes [27]. Another review of maternal anxiety reported that individualized interventions provided by experts, such as midwives or obstetricians, are effective for women with severe fear of childbirth and that hospital-based childbirth education is an effective intervention for pregnant women with anxiety [28]. In this study, the navigator was a midwife who conducted the patient navigation program based on extensive clinical experience with diverse delivery-related situations. This expertise enabled a multidimensional assessment of maternal anxiety by integrating psychological, environmental, and obstetric factors. In particular, the navigator promptly identified and addressed participants’ uncertainty about pregnancy and childbirth. When needed, she provided appropriate interventions and linked participants to resources, thereby connecting pregnancy, childbirth, and the postpartum period as a continuous process. Considering the multiple factors that contribute to maternal anxiety and their effects, prenatal interventions should provide education that addresses the information pregnant women need and should include supportive care. This study suggests that when nurses with clinical expertise provide educational interventions, practical advice, and explanations about the delivery room, childbirth preparation, and breathing techniques may help reduce anxiety among pregnant women approaching childbirth.

In this study, childbirth confidence increased after the intervention in the experimental group that participated in the prenatal navigation program, and the between-group difference in change scores was significant. However, the pretest difference in childbirth confidence between the two groups should be considered when interpreting this result. Pregnant women who were more interested in the patient navigation program, had greater concerns about childbirth, or had greater preparation needs may have been more likely to participate in the experimental group. These group characteristics may have contributed to the baseline difference in childbirth confidence. Although direct comparison is difficult because research on prenatal patient navigation programs remains limited, the present findings are consistent with previous studies showing increased childbirth confidence after experience-oriented prenatal programs conducted by dedicated nurses [5] or by midwives for primigravidas at 32 weeks of gestation or later [17].

This patient navigation program was structured around education that reflected individual needs, which is a key element of navigation interventions. At each session, individual interviews were conducted with the 28 pregnant women in the experimental group to identify weekly educational needs and concerns, and feedback and interventions were provided accordingly. Previous studies have also reported that educational programs reflecting pregnant women’s needs and incorporating delivery room tours for childbirth experience had positive effects on childbirth confidence [5,17]. Education that addresses individual needs and barriers may improve pregnant women’s understanding of the labor process and childbirth preparation, thereby supporting childbirth confidence. In addition to providing obstetric nursing care, the dedicated navigator continuously assessed participants’ psychological readiness for childbirth as a core component of the patient navigation program. Helping participants express anxiety and fear about childbirth and providing empathy and supportive interventions may also have contributed to increased childbirth confidence.

Previous studies have reported that spousal support positively affects childbirth confidence in pregnant women [20]. In this study, the educational content was provided as videos that participants were encouraged to watch with their spouses, although spousal participation was not mandatory. This optional spousal participation may have helped spouses understand the physical and emotional states of pregnant women and take a cooperative, active role in preparing for vaginal delivery. The resulting sense of connection with the spouse may have been associated with improved childbirth confidence. However, because spousal participation was not controlled, this possibility should be examined more systematically in future research. In addition, introducing community resources may have improved participants’ access to resources and strengthened their ability to make decisions independently, which may also have had a positive effect on childbirth confidence.

Overall, these findings support previous research indicating that a nurse-led postpartum patient navigation program improved participant satisfaction through continuity of care, an individualized approach, reliable advice, rapid responses to needs, and support for decision-making [14]. The findings are also consistent with previous studies suggesting that patient navigation for pregnant women can support prenatal visit scheduling and continuity of care, provide prenatal health education and breastfeeding support, and improve perinatal women’s healthcare experiences through links to pediatric care and maternal and community resources [7,15].

Therefore, if nurses with extensive delivery experience lead prenatal patient navigation programs in clinical practice, they may help strengthen self-care capacity and psychological stability among pregnant women by providing accurate education about specific pregnancy and childbirth situations. These effects may, in turn, help reduce anxiety and increase childbirth confidence. The program components—education reflecting individual needs, a delivery room experience, psychological support, introduction to community resources, and opportunities for spousal participation—may help strengthen the intervention. Differentiated prenatal navigation programs tailored to pregnant women at various gestational stages may also help reduce physical, psychological, and social discomfort during pregnancy and improve attitudes toward childbirth.

However, this study has several limitations. First, because it was conducted at a single hospital among primigravidas at 35 weeks of gestation or later, the findings may not be generalizable to pregnant women in other regions or healthcare settings. Second, because a nonequivalent control group pretest-posttest design without random assignment was used, selection bias cannot be excluded. In particular, the groups were not homogeneous in pretest childbirth confidence scores, and this baseline difference should be considered when interpreting the results. Despite these limitations, this study is meaningful because it empirically examined an expert-led prenatal patient navigation program for primigravidas in late pregnancy. The study planned and implemented a prenatal navigation program led by a qualified professional and evaluated its outcomes. Although the intervention focused on the period immediately before delivery, the findings suggest that this approach could be expanded into a continuous navigation program linking prenatal and postpartum care, thereby supporting individualized and continuous maternal health management. In conclusion, this 3-week navigation program led by a nurse navigator for primigravidas at 35 weeks of gestation or later was associated with a greater reduction in anxiety and a greater increase in childbirth confidence in the experimental group than in the control group.

This study showed that a 3-week, 3-session navigation program delivered by a nurse with midwifery qualifications to first-time pregnant women preparing for childbirth reduced anxiety and improved childbirth confidence, and that the program could be applied in clinical settings. These findings suggest that structured navigation interventions provided by nurses in clinical prenatal care settings are feasible and may serve as practical strategies to support psychological adaptation among first-time pregnant women. Therefore, this program can be considered a nursing intervention for prenatal and childbirth education settings and may be applied in clinical nursing practice as an integrated model that combines tailored education and emotional support. At the nursing education and policy levels, the role of prenatal navigation should be clarified, and strategies should be developed for implementing interventions based on nurses’ clinical expertise.

## Figures and Tables

**Table 1.** Contents of the pregnancy navigation program

Sessions	Navigation domain	Contents	Method	Time (minute)
1st week: understanding pregnancy	Personalized assessment of individual needs and barriers	▪ Rapport building	PPT and printed handouts	60
		- Introduction of researchers and program	Video	60
		▪ Checking Individual needs and obstacles		
		▪ Physical changes by week of pregnancy		
		▪ Education on pregnancy needs and support		
		▪ Q&A		
2nd week: experience the delivery room and understand the normal delivery	Experiential preparation for childbirth	▪ Review-based feedback	Experience, PPT, printed handouts	60
		▪ Experiencing the delivery room	Video	60
		-Tour of the delivery room		
		-Nonstress test		
		-Breathing, exercise, pushing		
		-Spousal support		
		▪ Normal labor process		
		-Characteristics of each stage of labor		
		-Normal fetal responses		
		▪ Preparing the birthing bag		
		▪ Education on childbirth-related needs and support		
		▪ Q&A		
3rd week: postpartal and neonatal care, breastfeeding	Postpartum adaptation support and community linkage	▪Review-based feedback	PPT and printed handouts	60
		▪Guidance for postpartum adaptation and self-management		
		▪ Newborn care		
		▪ Breastfeeding		
		▪ Community resource linkage		
		▪ Q&A		

PPT: PowerPoint; Q&A: question and answer.

**Table 2.** Homogeneity tests on the characteristics between groups (N=56)

Characteristics	Categories	n (%)	n (%) or mean±SD		χ^2^ or t or z	*p*
			Exp. (n=28)	Cont. (n=28)		
Age (year)	≤30	19 (33.9)	10 (35.7)	9 (32.1)	0.08	.778
	≥31	37 (66.1)	18 (64.3)	19 (67.9)		
	Mean±SD	31.6±2.56	31.8±2.56	31.4±2.59	0.57	.571
Education	≤Bachelor	49 (87.5)	23 (82.1)	26 (92.9)		.225
	≥Master	7 (12.5)	5 (17.9)	2 (7.1)		
Religion	Have	17 (30.4)	10 (35.7)	7 (25.0)	0.76	.383
	None	39 (69.6)	18 (64.3)	21 (75.0)		
Occupation	Yes	38 (67.9)	18 (64.3)	20 (71.4)	0.33	.567
	No	18 (32.1)	10 (35.7)	8 (28.6)		
Monthly income (10,000 KRW)	<500	33 (58.9)	19 (67.9)	14 (50.0)	1.85	.174
	≥500	23 (41.1)	9 (32.1)	14 (50.0)		
Gestational ages (week)	<36	35 (62.5)	15 (53.6)	20 (71.4)	1.91	.168
	≥36	21 (37.5)	13 (46.4)	8 (28.6)		
	Mean±SD	35.7±0.58	35.8±0.55	35.6±0.61	1.62	.110
Planned pregnancy	Yes	43 (76.8)	20 (71.4)	23 (82.1)	0.90	.342
	No	13 (23.2)	8 (28.6)	5 (17.9)		
View of normal delivery	Positive	50 (89.3)	25 (89.3)	25 (89.3)		>.999
	Negative	6 (10.7)	3 (10.7)	3 (10.7)		
Anxiety			43.93±7.39	40.68±9.47	1.43	.158
Childbirth confidence			33.18±4.47	37.68±6.39	–3.08^†^	.002

Cont.: Control group; Exp.: experimental group; KRW: Korean won (one million KRW is roughly 700 US dollars).

† Analyzed using the Mann-Whitney U test.

**Table 3.** Changes in anxiety and childbirth confidence between groups (N=56)

Variables	Groups	Mean±SD or median (IQR)			t or z	*p*	d or r
		Pretest	Posttest	Difference			
Anxiety	Exp. (n=28)	43.93±7.39	35.39±6.30	–8.54±7.40	–6.15	<.001	0.76
	Cont. (n=28)	40.68±9.47	44.50±7.63	3.82±7.6			
Childbirth confidence	Exp. (n=28)	33.00 (33.00 to 34.00)	39.00 (37.00 to 42.00)	6.50 (4.25 to 10.00)	–5.70^†^	<.001	0.781
	Cont. (n=28)	37.00 (34.00 to 39.75)	33.50 (29.00 to 39.25)	–2.50 (–6.00 to –0.25)			

Cont.: Control group; Exp.: experimental group; IQR: interquartile range.

† Analyzed using the Mann-Whitney U test.
